# The molecular cloning and clarification of a photorespiratory mutant, oscdm1, using enhancer trapping

**DOI:** 10.3389/fgene.2015.00226

**Published:** 2015-07-03

**Authors:** Jinxia Wu, Zhiguo Zhang, Qian Zhang, Xiao Han, Xiaofeng Gu, Tiegang Lu

**Affiliations:** Biotechnology Research Institute/National Key Facility for Genetic Resources and Gene Improvement, The Chinese Academy of Agricultural SciencesBeijing, China

**Keywords:** rice, serine hydroxymethyltransferase, photorespiratory, mitochondria, hydrogen peroxide

## Abstract

Enhancer trap systems have been demonstrated to increase the effectiveness of gene identification in rice. In this study, a chlorophyll-deficient mutant, named *oscdm1*, was screened and characterized in detail from a T-DNA enhancer-tagged population. The *oscdm1* plants were different from other chlorophyll-deficient mutants; they produced chlorotic leaves at the third leaf stage, which gradually died with further growth of the plants. However, the *oscdm1* plants were able to survive exposure to elevated CO_2_ levels, similar to photorespiratory mutants. An analysis of the T-DNA flanking sequence in the *oscdm1* plants showed that the T-DNA was inserted into the promoter region of a serine hydroxymethyltransferase (SHMT) gene. OsSHMT1 is a key enzyme that is ubiquitous in nature and structurally conserved across kingdoms. The enzyme is responsible for the interconversion of serine and glycine and is essential for cellular one-carbon metabolism. Full-length *OsSHMT1* complemented the *oscdm1* phenotype, and the downregulation of *OsSHMT1* in wild-type plants by RNA interference (RNAi) produced plants that mimicked the *oscdm1* phenotype. GUS assays and quantitative PCR revealed the preferential expression of *OsSHMT1* in young leaves. TEM revealed serious damage to the thylakoid membrane in *oscdm1* chloroplasts. The *oscdm1* plants showed more extensive damage than wild type using an IMAGING-PAM fluorometer, especially under high light intensities. OsSHMT1-GFP localized exclusively to mitochondria. Further analysis revealed that the H_2_O_2_ content in the *oscdm1* plants was twice that in wild type at the fourth leaf stage. This suggests that the thylakoid membrane damage observed in the *oscdm1* plants was caused by excessive H_2_O_2_. Interestingly, *OsSHMT1*-overexpressing plants exhibited increased photosynthetic efficiency and improved plant productivity. These results lay the foundation for further study of the *OsSHMT1* gene and will help illuminate the functional role of OsSHMT1 in photorespiration in rice.

## Introduction

Leaves are the most important organs for photosynthesis in plants; indeed, the photosynthetic efficiency of leaves determines crop productivity. Improving photosynthesis in leaves is a reliable method for breeding cultivars with high photosynthetic efficiency (Govaerts et al., 1996; Zhang et al., 2009). The key to this method is cloning and analyzing the function of genes involved in leaf photosynthesis. Leaf color mutants are ideal genetic materials for studying the molecular mechanisms that regulate leaf photosynthesis and photorespiration in plants. To date, a considerable number of leaf color mutations have been reported in rice (http://www.shigen.nig.ac.jp/rice/oryzabaseV4), and some of them have been cloned successfully. For example, *OsCHLH* was the first gene from rice to be identified from a T-DNA insertion mutant using gene trapping methods. *OsCHLH* encodes the OsChlH subunit of magnesium chelatase, and its mutation produces plants that lack chlorophyll in their thylakoids (Jung et al., 2003; Goh et al., 2004). *YGL1* encodes a chlorophyll synthase; its mutation reduces chlorophyll accumulation and delays chloroplast development (Wu et al., 2007). *Chl1* and *Chl9* encode key enzymes for chlorophyll synthesis and chloroplast development, respectively. Ultrastructural analyses have revealed that the grana of Chl1 and Chl9 mutants are poorly stacked, resulting in underdeveloped chloroplasts (Zhang et al., 2006). *OsPPR1* encodes a pentatricopeptide repeat protein that may play an essential role in chloroplast biogenesis; its mutant exhibits typical phenotypes of chlorophyll-deficient plants (Gothandam et al., 2005). *OsHAP3* regulates normal chloroplast development and chlorophyll biosynthesis, and its mutant has pale green leaves (Miyoshi et al., 2003).

The mutants and genes described above are mostly related with chlorophyll synthesis or degradation in rice. In recent years, several genes from other pathways affecting leaf color have been identified in monocotyledonous plants. For example, several tie-dyed mutants, including tie-dyed1, tie-dyded2, camoflauge1, psychedelic, and sucrose export defective1, have been described that show chlorotic/variegated leaves and carbon hyperaccumulation in their leaf blades (Braun et al., 2006; Baker and Braun, 2007, 2008; Ma et al., 2008; Huang et al., 2009; Slewinski and Braun, 2010; Slewinski et al., 2012; Baker et al., 2013). Tie-dyed1 (Tdy1) is a novel transmembrane protein that is present only in grasses. Tdy1 functions in carbon partitioning by promoting phloem loading; accordingly, it is expressed exclusively in the phloem cells of both source and sink tissues. The *tie-dyed2* (*tdy2*) mutant exhibits variegated green and yellow leaves. Tie-dyed2 is a callose synthase that functions in vein development and affects symplastic trafficking within the phloem of maize leaves. Several other maize leaf color genes, including *Oil Yellow1* (*Oy1*), those in certain lesion mimic mutants, and *Iojap* (*Ij*), have also been cloned. The semi-dominant *Oy1* mutants of maize are deficient in the first committed step of chlorophyll biosynthesis: the conversion of protoporphyrin IX to magnesium protoporphyrin IX. The *Oy1* gene encodes subunit I of magnesium chelatase (Sawers et al., 2006). The lethal leaf spot 1 lesion mimic locus of maize (*ZmLls1*) encodes pheophorbide a oxygenase. This locus corresponds to gene At3g44880 on chromosome 3 of *Arabidopsis thaliana* (Gray et al., 2002; Yang et al., 2004a). *Iojap* (*ij*) is a recessive striped mutant of maize in which plastid development is locally altered in a position-dependent manner on the leaves; *ij*-affected plastids can be transmitted to progeny even when the function of the nuclear gene is restored. The *ij* mutant is characterized by a number of independent transposon insertion mutations created using Robertson's Mutator. The *Ij* gene encodes a 24.8-kDa protein with no significant sequence similarity to proteins in public databases (Han et al., 1992; Byrne and Taylor, 1996; Silhavy and Maliga, 1998). In addition to these leaf color mutants, the barley mutants *tigrina-d* (Lee et al., 2003; Khandal et al., 2009) and *chlorina* have been well characterized (Knoetzel et al., 1998; Krol et al., 2009).

Plant photorespiration is an essential prerequisite for oxygenic photosynthesis. This metabolic repair pathway bestrides four compartments and requires several metabolite transporters for pathway function and the well-studied enzymatic steps of the core photorespiratory cycle (Eisenhut et al., 2014). Photorespiration dissipates excess photochemical energy to provide protection against oxidative damage under stressful conditions in which CO_2_ assimilation is reduced. In addition, photorespiration provides metabolites for protection against stress, including glycine for the synthesis of glutathione (Lakshmanan et al., 2013). Mutants with photorespiration pathway deficiencies exhibit chlorotic and lethal phenotypes when grown in ambient CO_2_ (Somerville and Ogren, 1981; Jamai et al., 2009). The enzymatic steps involved in the photorespiratory pathway have been well established. For example, mitochondrial serine hydroxymethyltransferase (SHMT) has been reported to be an essential component of the pathway; it plays an important role in one-carbon metabolism. Specifically, the enzyme produces N^5^,N^10^-methylene tetrahydrofolate (THF) glycine (Gly) from Ser and THF, and generates one-carbon units for cellular use (Mouillon et al., 1999). Seven SHMT genes have been found in *Arabidopsis* and other species. The *Arabidopsis* SHMT mutant *shm1* was one of the first photorespiratory mutants described by Somerville and Ogren (1981); the affected gene was later cloned by map-based cloning (Voll et al., 2006). AtSHMT1 was reported to play a critical role in controlling cell damage caused by abiotic stress, whereas its mutant *shm1-1* has a conditional lethal photorespiratory phenotype (Moreno et al., 2005). Thus, SHMT has acquired a new or altered function. A soybean (*Glycine max* L. Merr.) SHMT conferring resistance to nematodes plays an important role in plant resistance mechanisms against the pathogen (Liu et al., 2012).

T-DNA tagging methods, including gene trapping, promoter trapping, and enhancer trapping, have been shown in rice to be more effective and faster than positional cloning. These techniques are also advantageous when dealing with early embryogenic or gametophytic lethality. Several genes have been cloned by gene trapping or enhancer trapping, including *OsCHLH* (Jung et al., 2003), *Wda1* (Jung et al., 2006), *UDT1* (Jung et al., 2005), *Rip1* (Han et al., 2006), and *MIT* (Bashir et al., 2011). We previously reported the establishment of a large T-DNA enhancer trapping population (Yang et al., 2004b; Peng et al., 2005; Wan et al., 2009). The vector used to establish the pool carried a minimal cauliflower mosaic virus (CaMV) 35S promoter containing only the TATA box and transcription start site, which was placed adjacent to the T-DNA border. Only when the insertion site is next to an enhancer can the minimal promoter drive reporter gene expression. The GAL4/VP16-UAS enhancer trap was used in our insertion pool; this system improves the efficiency of enhancer trapping greatly. Insertion of the T-DNA into a gene in the proper orientation enables fusion between the endogenous gene and the GUS reporter gene; thus, T-DNA-tagged lines can be verified by a GUS assay. Screening of our insertion lines revealed that 40% were positive for GUS in the leaves or seeds (Peng et al., 2005).

To understand the molecular mechanism controlling leaf color, we first screened for leaf color mutants in the T_1_ generation. Leaf color phenotypes co-segregated with GUS staining in nine independent lines. One line, *oscdm1*, showed co-segregation between its mutant phenotype and the T-DNA; thus, it was selected for further analysis. The *oscdm1* plants had yellow-green chlorotic leaves; this trait was found to be controlled by a single nuclear gene. The *oscdm1* plants displayed a lethal phenotype under natural conditions. Amplification of the T-DNA insertion-flanking sequence by PCR-based genome walking and subsequent sequencing resulted in the identification of an SHMT gene, *OsSHMT1*, which is predicted to encode the largest subunit of SHMT. The mutant phenotype was complemented by transformation with the wild-type gene, and plants in which *OsSHMT1* expression was reduced by RNA interference (RNAi) exhibited the *oscdm1* mutant phenotype. Moreover, the *oscdm1* plants exhibited extensive thylakoid membrane damage compared to wild type, especially at high light intensities. This damage may have resulted from excessive H_2_O_2_ levels. The identification of *OsSHMT1* using enhancer trapping and the characterization of *oscdm1* will aid future studies of the exact role of OsSHMT1 in photorespiration in rice.

## Materials and methods

### Plant materials

The enhancer trapping population used in the present study for the mutant screen was described previously (Peng et al., 2005). The functional regions of the enhancer trap vector pFX-E24.2-15R are shown in Additional File 1: Figure S1. Within the T-DNA, the reporter gene was comprised of −48CaMV, *GAL*4/*Vp*16, 6×UAS, BoGUS, and EGFP; the reporter was located at the right border (RB) of the T-DNA. The minimal promoter −48CaMV cannot drive expression of the reporter gene alone; however, when the reporter gene is inserted into a gene or nearby enhancer it can be activated by the minimal promoter with the help of these enhancer elements.

### Leaf color mutant isolation

About 30 T_1_ seeds per line were sterilized in 1% sodium hypochlorite for 30 min, washed with running tap water, and then imbibed in tap water for 3 days at 20°C. The seeds were then sown on a plastic net floating on nutrient fluid (Yoshida et al., 1976). The pH was adjusted every 2 days, with replacement of the nutrient fluid every week. The plants were grown for 14–30 days under greenhouse conditions, with natural sunlight and a minimum nighttime temperature of 25°C.

### Histochemical staining for GUS activity in plant tissues

GUS assays were performed essentially as described by Jefferson et al. (1987). Rice tissues were removed, transferred to Eppendorf tubes containing GUS staining solution (50 mM sodium phosphate, pH 7.0, 10 mM EDTA, 0.1% Triton X-100, 1 mg/ml X-gluc, 0.1 mM potassium ferricyanide, and 10% methanol), and incubated at 37°C for 10–12 h. The staining solution was then removed and the tissues were stored in 70% ethanol. The stained tissues were examined under a zoom stereomicroscope (Nikon SMZ1000; Tokyo, Japan) and photos were taken with a digital camera (Nikon DXM1200F).

### Amplification and analysis of the T-DNA flanking sequences

Genomic DNA from the enhancer trap mutants was isolated using an improved version of the CTAB method (Murray and Thompson, 1980), and the T-DNA left border (LB) flanking regions were rescued using a modified version of the PCR-based genome walking protocols of Siebert et al. (1995) and Sallaud et al. (2003). The method consisted of three steps: digestion of genomic DNA using a blunt-end restriction enzyme and ligation of an asymmetrical adaptor, amplification by PCR using primers specific for the T-DNA and the adaptor, respectively, and successive PCR using two nested, specific primers. The adaptor (ADAR, 50 mM) was prepared by heating a mixture comprised of equal volumes of the complementary oligonucleotides ADAR1 (100 mM) and ADAR2 (100 mM) to 80°C for 10 min, and then allowing it to cool gradually to room temperature for annealing. The specific primers for the adaptor were APR1 (20 mM) and APR2 (20 mM); the specific primers for the T-DNA LB in pFX-E24.2-15R were LB1 (20 mM) and LB2 (20 mM). The distance between the LB2 binding site and the LB was about 180 bp. Unwanted amplification resulting from the two adaptors was inhibited by the asymmetry of the adaptor and its NH_2_-blocked internal 3′ end; the second round of nested PCR also reduced non-specific amplification reactions (Siebert et al., 1995). Each DNA sample (40–100 ng) was digested using *Dra*I, *Eco*RV, or *Pvu*II (2 U; Takara, Dalian, China) and ligated with ADAR (0.1 ml) using T4 DNA ligase (70 U; Takara) at 25°C in a total volume of 10 μl (1 μl of 10× ligase buffer). After digestion/ligation for 10–12 h, aliquots (2 μl each) were used for the first round of PCR (MJ Research PTC-100 DNA Engine; MJ Research Inc., Quebec, Canada) in a total volume of 20 μl (0.3 ml of LB1, 0.3 ml of APR1, 2 U of Takara r-Taq, 100 mM dNTPs, and 2 μl of 10× PCR buffer). The program was as follows: 3 min at 94°C followed by 35 cycles of 94°C for 30 s, 67°C for 45 s, and 72°C for 150 s, with a final elongation step at 72°C for 5 min. Next, 2 μl of a 1:50 dilution of the product was used for nested PCR in a total volume of 20 μl (0.5 μl of LB2, 0.5 μl of APR2, 0.8 U of r-Taq, 200 mM dNTPs, and 2 μl of 10× PCR buffer) under the same conditions. All of the products from the second round of PCR were loaded onto a 1.5% agarose gel for electrophoresis. Distinct DNA bands were recovered for direct sequencing using an ABI 3730xl DNA analyzer (Applied Biosystems, Life Technologies, Carlsbad, CA, USA), which has a sequencing capability of >1 kb. LB2 was used as the primer for sequencing according to the protocol included with the BigDye Terminator v3.1 Cycle Sequencing Kit (Life Technologies).

### Co-segregating genotype analysis of *oscdm1* by PCR

An analysis of the *oscdm1* plants was performed by PCR using two gene-specific primers flanking the insertion site (P1 and P2) and another T-DNA LB-specific primer (P3). PCR was carried out in a total volume of 20 μl containing 20 ng of plant DNA, 10× PCR buffer, 0.2 mM dNTPs, 0.5 U of rTaq polymerase, and 1 mM each primer. The DNA was denatured at 95°C for 4 min, followed by 35 cycles of 94°C for 1 min, 58°C for 1 min, and 72°C for 2 min. The products were loaded onto a 1.5% agarose gel for electrophoresis.

### *OsSHMT1* cDNA amplification, complementation analysis, and overexpression line construction

Total RNA was extracted using TRIzol reagent (Invitrogen, Carlsbad, CA, USA) from wild-type plants. The RNA was quantified using a UV spectrophotometer (Beckman DU 800 Series UV/VIS Spectrophotometer; Beckman Coulter Inc., Brea, CA, USA). First-strand cDNA was synthesized by reverse transcription using a cDNA synthesis kit (Takara) in a total volume of 20 μl containing 1 μg of total RNA, 10 ng of oligo(dT)_14_ primer, 2.5 mM dNTPs, 1 μl of AMV, and 0.5 μl of RNAsin. Amplification of the full-length cDNA sequence of *OsSHMT1* (1542 bp) was performed in a total of 20 μl containing a 1/20 aliquot of the cDNA reaction, 0.5 μM gene-specific primers (OP1, which contained a *Sal*I digestion site, and OP2, which contained a *Sma*I digestion site), 10 mM dNTPs, 1 U of rTaq DNA polymerase, and 2 μl of 10× reaction buffer. The reaction protocol was as follows: denaturation at 94°C for 3 min followed by 25 cycles of 94°C for 30 s, 60°C for 45 s, and 72°C for 1 min, with a final step at 72°C for 10 min. An aliquot (1 ţl) of the reaction was loaded onto a 1.5% agarose gel (regular; Biowest, Barcelona, Spain) and analyzed by electrophoresis. The PCR product was cloned into the pEASY-Blunt simple cloning vector (Beijing TransGen Biotech Co., Ltd., Beijing, China) and sequenced. For the complementation construct, full-length *OsSHMT1* cDNA was cloned into pCambia23A carrying the actin promoter to generate Actin::OsSHMT1. The constructs were introduced into *Agrobacterium tumefaciens* (strain EHA105) by electroporation and *Agrobacterium*-mediated transformation was performed using vigorously growing *oscdm1* calli derived from a segregating population. OsSHMT1-overexpressing (OV) lines were constructed by transforming Actin::OsSHMT1 into Nipponbare calli.

### Generation of pUbi-RNAi309-SHMT1 transgenic rice

DNA corresponding to 307 bp of the 3′ end of *OsSHMT1* was amplified using the primers P6 and P7. The product was cloned into the pEASY-Blunt simple cloning vector (Beijing TransGen Biotech Co., Ltd.) and sequenced. The vector was inserted into pTCK309 using the *Sac*I, *Spe*I, *Kpn*I, and *Bam*HI sites in inverted orientations. The pUbi-RNAi309-SHMT1 construct was transferred to *Agrobacterium* strain AGL1 by electroporation and then introduced into wild-type rice calli through *A. tumefaciens*-mediated transformation. The regenerated T_0_ plants were grown in a paddy field at the Chinese Academy of Agricultural Sciences in Beijing, China. Leaves of the pUbi-RNAi309-SHMT1 transgenic plants showing the *chlorina* phenotype were selected and analyzed.

### Reverse transcription (RT)-PCR and quantitative real-time RT-PCR (qRT-PCR)

To study the *OsSHMT1* expression level, total RNA was extracted using TRIzol reagent (Invitrogen) from the leaves of wild-type and *oscdm1* plants. Total RNA (2 μg) from each sample was reverse-transcribed with oligo(dT) primer and PrimeScript RT Enzyme (Takara) according to the manufacturer's instructions. For RT-PCR, the primers used to amplify *OsSHMT1* were Q1f and Q1R, while the primers used to amplify actin were Actinf and Actinr. The PCR conditions were as follows: preincubation at 94°C for 2.5 min, then 30 cycles of 94°C for 30 s, 52°C for 30 s, and 72°C for 1 min. qRT-PCR was performed using an iQTM5 Muticolor Real-Time PCR Detection System (Bio-Rad, Hercules, CA, USA) with SYBR® Green Real-Time PCR Master Mix (Life Technologies). The primers used to amplify *OsSHMT1* were qF and qR.

### Transmission electron microscopy (TEM)

Leaves were harvested from wild-type and *oscdm1* plants at the third leaf stage that had been grown in a greenhouse under a medium light intensity (~150 μmol of photons/m^2^s). Leaf sections were fixed in 2% glutaraldehyde and further fixed in 1% OsO_4_. The tissues were then stained with uranyl acetate, dehydrated in ethanol, and embedded in Spurr's medium prior to thin sectioning. The samples were then stained again and examined with a JEOL 100 CX electron microscope (JEOL Ltd., Tokyo, Japan). The total chlorophyll content was determined spectrophotometrically according to the method of Arnon and Whatley (1949). Leaves (300 mg) were ground to a powder in liquid nitrogen and then transferred to a 15-ml Falcon tube. Next, 5 ml of 80% acetone were added to the tube and the mixture was thoroughly combined then left in the dark overnight. Centrifugation was performed at 4°C for 15 min (3000 rpm). The supernatant was transferred to a new centrifuge tube and the chlorophyll absorbance (hereafter termed A) was measured by spectrophotometry. The chlorophyll concentrations were calculated as follows (using 80% acetone as a blank control): C_*a* + *b*_ (mg/g) = [8.026A_663_ + 20.206A_645_] × V/1000 × W, where V is the volume of the extract (ml) and W is the fresh leaf weight (g).

### Chlorophyll fluorescence measurement

The middle portion of the first leaves from wild-type and *oscdm1* plants at the third leaf stage were collected and used to measure the leaf photosynthetic efficiency. Chlorophyll fluorescence measurements were performed with an IMAGING-PAM 2000 chlorophyll fluorometer (Walz, Effeltrich, Germany). A small piece of the mutant leaf blade was removed and placed in the well of a PCR plate filled with water; after 15 min of dark preadaptation, the measurements were performed. Changes in the chlorophyll fluorescence of the chlorophyll-deficient mutants were documented based on F_t_, the steady level of chlorophyll fluorescence; F_v_/F_m_ = (F_m_−F_0_)/F_m_, the maximum quantum yield of photosystem (PS)II photochemistry; Φ_PSII_ = (F′_m_−F)/F′_m_, the effective quantum yield of PSII; qP = (F′_m_−F)/(F′_m_ −F′_0_), photochemical quenching; and qN = 1−([F′_m_−F′_0_]/[F_m_ − F_0_]), non-photochemical quenching. The measurements were recorded after 5 min under actinic light. F′_0_ was calculated as F′_0_ = F_0_/(F_v_/F_m_+F_0_/F_m_) because of the limitations of the fluorometer.

### Blue native (BN) test

BN polyacrylamide gel electrophoresis for the isolation of membrane protein complexes in an enzymatically active form was performed mostly as described (Schagger et al., 1994; Yan et al., 2007). Thylakoids were resuspended in medium A (25 mM Bis-Tris HCl, pH 7.0, 20% [w/v] glycerol, and 0.25 mg/ml Pefabloc) to a final concentration of 0.5 mg/ml chlorophyll, and an equal volume of 2% (w/v) dodecyl β-D-maltoside (Sigma, St. Louis, MO, USA), freshly prepared in medium A, was added. The thylakoids were then solubilized on ice for 1 min and centrifuged at 18,000 × g at 4°C for 12 min. The supernatant was supplemented with a 1/10 volume of buffer (100 mM Bis-Tris HCl, pH 7.0, 0.5 M ε-amino-n-caproic acid, 30% [w/v] sucrose, and 50 mg/ml Serva Blue G) and loaded on a gel containing a 5–12% gradient of acrylamide in the separation gel. Electrophoresis was performed [using the Hoefer Mighty Small system from Amersham Biosciences (Little Chalfont, UK)] at 0°C for 3.5 h by gradually increasing the voltage from 75 to 200 V. The thylakoids photosynthetic complexes were separated.

### Measurement of the H_2_O_2_ levels

The H_2_O_2_ levels in the plants were measured according to the method of Dagmar et al. (2001). Briefly, 500 mg of leaves were homogenized in a cold mortar with a pestle and 0.2 g of silicon dioxide in pre-cooled acetone (5 ml). The homogenate was centrifuged at 12,000 × g for 5 min, after which 1 ml of the supernatant was mixed with 0.1 ml of 5% Ti(SO_4_)_2_ and 0.2 ml of 19% ammonia. After the formation of a precipitate, the reaction mixture was centrifuged at 12,000 × g for 5 min. The resulting pellet was dissolved in 3 ml of 2 M H_2_SO_4_ and the absorbance of the solution was recorded at 415 nm. The H_2_O_2_ content was calculated according to a standard curve of H_2_O_2_ ranging from 0 to 10 mM.

### Photosynthetic parameter measurements

The photosynthetic rate, transpiration rate, and stomatal conductance in wild-type and OsSHMT1- OV plants were measured at the full heading stage using a portable photosynthetic LCPRO^+^ instrument (ADC BioScientific, Hoddesdon, UK) with the following settings: 500 mmol/s flow velocity, 30°C leaf chamber, and 1800 umol/s light quantum flux density.

### Phylogenetic analysis

The BLAST search program (http://blast.ncbi.nlm.nih.gov/) was used to identify homologs of OsSHMT1 using its amino acid sequence as the query. The resulting sequences were aligned using ClustalX1.83 in multiple alignment modes, and a neighbor-joining phylogenetic tree was generated using MEGA 4.0 (Tamura et al., 2007). The bootstrap values for nodes in the phylogenetic tree were from 1000 replications. The handling gap option was pairwise deletion; the numbers at the branch points indicate the bootstrap values. The peptide identities among the different proteins were calculated using GeneDoc software. The OsSHMT1 nucleotide/amino acid sequence has been deposited in the NCBI database with accession number: Os03g0738400. The OsSHMT1 full cDNA clone AK063056 has been deposited in KOME.

### Subcellular localization

Full-length *OsSHMT1* cDNA was amplified using the primers Fp1 and Rp1, after which the product was cut with *Sma*I and *Xba*I and then fused with PAN580. The 35S::OsSHMT1-GFP expression construct was transfected into rice protoplasts as described previously (Zhang et al., 2011). Briefly, for each sample, 10 μg of plasmid DNA were mixed with 200 μl of protoplasts (~1 × 10^6^ cells). Freshly prepared polyethylene glycol solution [220 μl; 40% (w/v) polyethylene glycol 4000, 0.2M mannitol, and 0.1 M CaCl_2_] was added, and the mixture was incubated at room temperature for 20 min in the dark. After incubation, 1 ml of W5 solution was added slowly to the samples. The resulting solution was mixed well by gently inverting the tube, and the protoplasts were pelleted by centrifugation at 1500 rpm for 3min. The protoplasts were resuspended gently in 1 ml of W5 solution. Finally, the protoplasts were transferred to multi-well plates and cultured under light or in the dark at room temperature for 16 h. MitoTracker® Red CMXRos was purchased from Invitrogen. The samples were observed with a confocal laser scanning microscope (Leica TCS SP5; Leica Microsystems, Wetzlar, Germany).

### Statistical analysis

All the experiments were carried out at least in triplicate. The values shown in the figures are mean values ± SD. For multiple comparisons, means were compared by one-way analysis of variance and Duncan's multiple range test with a 5% level of significance.

### Primer sequence

All the primers for gene cloning, complementation analysis, over-expression construction, sub-cellular localization, reverse transcription (RT)-PCR and quantitative real-time (qRT-PCR) and etc in our paper are listed in Table S1.

## Results

### Isolation of chlorophyll-deficient mutants from the T-DNA insertion population

**Figure 1:**
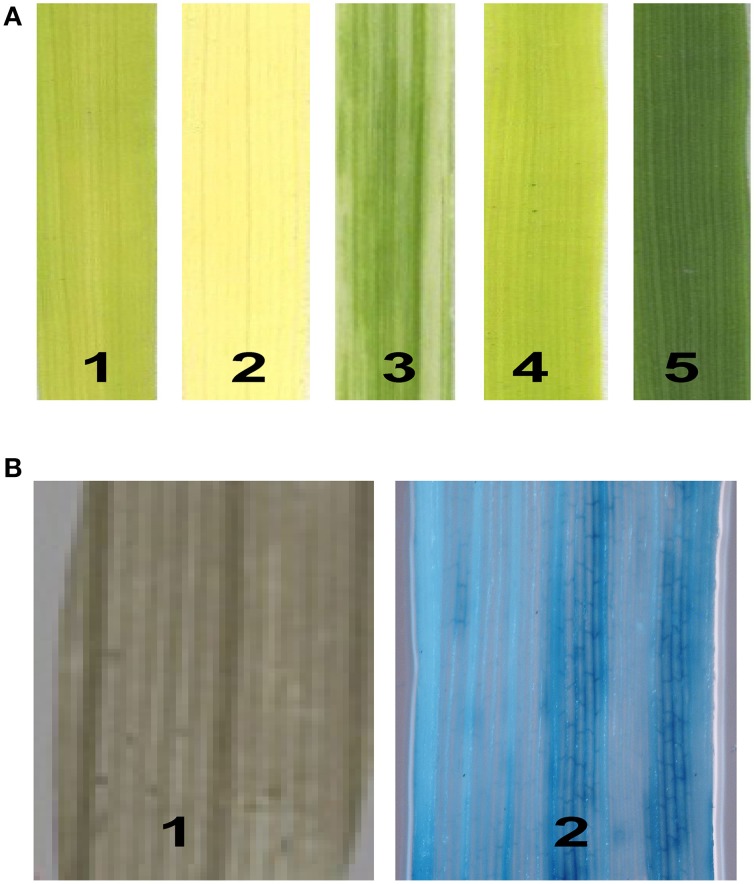
**Isolation of chlorophyll-deficient mutants from the T-DNA-mutagenized population. (A)** The phenotypes of the chlorophyll-deficient mutants: 1, yellow; 2, albino; 3, stripe; 4, pale green; and 5, wild type. **(B)** GUS expression patterns in the leaves: 1, negative; 2, vascular tissue-specific.

To identify new genes modulating the photosynthetic mechanism in rice, we conducted a genetic screen of an enhancer trapping population containing more than 10,000 individual lines. In total, 420 lines with leaf blade color defects were identified, including 251 albino, 132 pale green, 35 chlorotic (yellow), 25 stripe, and 13 zebra. The mutant lines varied in phenotype from albino to pale green according to the degree of chlorophyll deficiency (Figure 1A). Of the 420 leaf color mutants tested, 34 lines were positive for GUS staining. However, only in nine lines were all of the mutants positive for GUS staining. These lines were selected for further study. To test for the co-segregation of GUS staining and the hygromycin resistance gene *HptII* in the T_1_ generation, we amplified DNA from each mutant line using *HptII*-specific primers. The results were consistent, as expected, for all mutants of the nine lines. The GUS expression patterns in the mutant leaves are shown in Figure 1B.

### Analysis of T-DNA insertion sites in the rice genome and prediction of tagged genes

PCR-based genome walking has been shown to be a highly efficient way of isolating T-DNA flanking sequences (Peng et al., 2005). Using this technique, 10 bands were amplified from the nine mutant lines. Of all of the bands sequenced, only six contained rice genomic sequences. The other four contained vector sequences, indicating border read-through or the termination of sequencing. Of the 10 flanking sequences rescued, six sequences originating from five mutants showed a unique hit to rice artificial chromosome (BAC/PAC) clones based on homology searches against sequences obtained from the NCBI and KOME databases. Of the six sequences, four flanking sequences were identified in genic regions and the other two in intergenic regions, based on a public annotation database for the rice genome. The locations of the T-DNA insertions are shown in Table 1.

**Table 1:** **Locations of the T-DNA insertion and tagged putative genes**.

**Line**	**GenBank accession number**	**Insertion site**	**BLASTn *E*-value**	**Chr**.	**Insertion region (putative genic or intergenic)**	**Localization of putative protein**
A33	AL731638	10450	2E-33	4	Intergenic region	
A167	AP002837	46557	9E-07	6	ORF of a putative Fe-SOD gene	Chloroplast
P239	AC117988	110492	9E-07	3	ORF of a putative glycine hydroxymethyltransferase gene	Mitochondrion
A304	AP004809	45787	3E-07	6	Intergenic region	
A401-1	AC125782	158205	3E-07	11	ORF of a putative GTP-binding gene	Chloroplast
A401-2	AP004182	53463	1E-17	7	ORF of a putative TPR-like domain-containing gene	No distinct localization

*ORF, open reading frame; SOD, superoxide dismutase; TPR, tetratricopeptide repeat*.

### Identification of a mutation in the SHMT promoter

Sequence analyses using the NCBI database identified one line, P239, also named *oscdm1*, as having a T-DNA insertion upstream of a gene with high homology to SHMT genes from several plant species. *OsSHMT1* encodes the largest subunit of SHMT. *OsSHMT1* is 3962 bp in length and consists of 16 introns and 17 exons, with a complete coding sequence of 1542 bp encoding 513 amino acids and a molecular mass of 56.3 kDa; it encodes a putative serine methyltransferase domain, which spans from residues 51–449 at the C-terminus (Figure S2).

### Characterization of the *oscdm1* mutant

**Figure 2:**
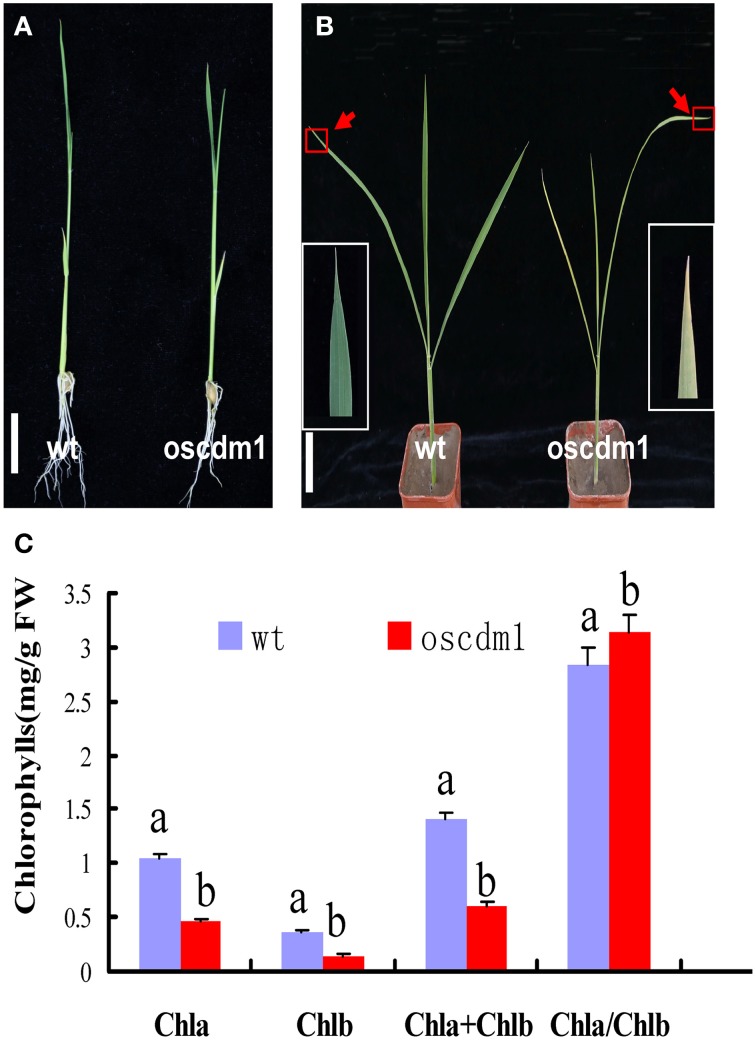
**Phenotype comparison of** ***oscdm1*** **and wild-type plants. (A)** Phenotypes of 10-day-old wild-type and mutant seedlings (bar = 1 cm). **(B)** Phenotypes of 1-month-old wild-type and mutant seedlings (bar = 1 cm). Red boxes denote the tips of the mutant and wild-type leaves. The insets show magnified views of the areas in the red boxes. **(C)** Measurement of the chlorophyll content in the leaves of 1-month-old wild-type and *oscdm1* mutant plants. The data are the means ± standard deviation (SD). Bars represent the SDs (*n* = 5). Data were compared by one-way analysis of variance and Duncan's multiple range test. Different letters (a – b) indicate significant differences (P, 0.05) between lines. Chla, chlorophyll *a*; Chlb, chlorophyll *b*.

There was no significant difference between 10-day-old (first leaf stage) *oscdm1* and wild-type plants (Figure 2A). However, the *oscdm1* plants appeared chlorotic at the third leaf stage (Figure 2B), and they gradually died off from the fourth leaf stage onward. We measured the chlorophyll contents of both the *oscdm1* plants and wild-type plants at the third leaf stage. The ratio of chlorophyll *a* to chlorophyll *b* in the wild-type plants was about 3.5. The chlorophyll *a*, chlorophyll *b*, and total chlorophyll contents in the *oscdm1* plants at the third leaf stage were drastically lower than in the controls (Figure 2C). These results suggest that the chlorotic phenotype of the *oscdm1* seedlings was probably caused by a reduction in the total chlorophyll content, rather than a reduction in a particular pigment.

**Figure 3:**
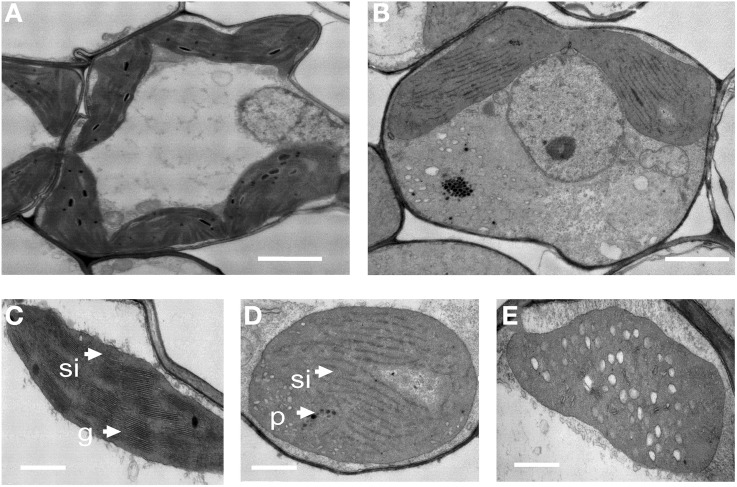
**Transmission electron microscopic images of chloroplasts from 1-month-old wild-type and** ***oscdm1*** **plants. (A)** Wild-type mesophyll cells. **(B)** The mesophyll cells of *oscdm1* plants. **(C)** Wild-type chloroplasts. **(D,E)** The chloroplasts of *oscdm1* plants. The chloroplasts of the wild-type plants had abundant, well-ordered membrane stacks, whereas the chloroplasts of the *oscdm1* mutant had almost no normal stacked membranous structures or grana **(D)**. The mutant chloroplasts also exhibited vacuolation **(E)**, p, plastoglobule; g, grana stack; sl, stroma lamellae; v, vacuolation. Bar = 1 μm in **(A,B)**, and 100 nm in **(C–E)**.

Light microscopic observations of cross-sections of a *chlorina* mutant leaf blade did not show any significant change in the size or number of mesophyll cells (data not shown). To examine whether the lack of chlorophyll in the *oscdm1* plants was accompanied by ultrastructural changes in the chloroplasts, we compared the ultrastructure of chloroplasts in the *oscdm1* and control plants at the third leaf stage using TEM. The analysis revealed no great change in chloroplast structure at the first leaf stage between the wild-type and *oscdm1* plants; however, at the third leaf stage, substantial changes in the number of chloroplasts, lamellar structures, and organization of the grana in the *oscdm1* chloroplasts (Figures 3B,D) were noted compared with wild type (Figures 3A,C). In addition, the mutant chloroplasts exhibited vacuolation (Figure 3E). The BN test was used to assess thylakoid membrane integrity. A BN gel analysis of the thylakoid membrane showed some differences in five bands between the mutant and wild-type plants; in particular, bands III and IV were slightly more diffuse in the mutant plants. We concluded that the light harvesting complexes (LHCs) in the thylakoid membrane were altered and that chlorophyll synthesis was affected in the mutants beginning at the third leaf stage (Figure S3).

### Co-segregation of the T-DNA and GUS activity with the *chlorina* phenotype

The *oscdm1* plants appeared chlorotic at the third leaf stage. These plants eventually died both under greenhouse conditions and in a paddy field because of insufficient photosynthesis. In the T_2_ generation, 80 plants were wild type and 30 were *oscdm1*. In the T_3_ generation, a heterozygous T_2_ plant generated 50 wild-type plants and 16 mutants, in accordance with our expectations for a single recessive locus. All of the chlorotic plants were GUS-positive, whereas only some of the wild-type plants were.

**Figure 4:**
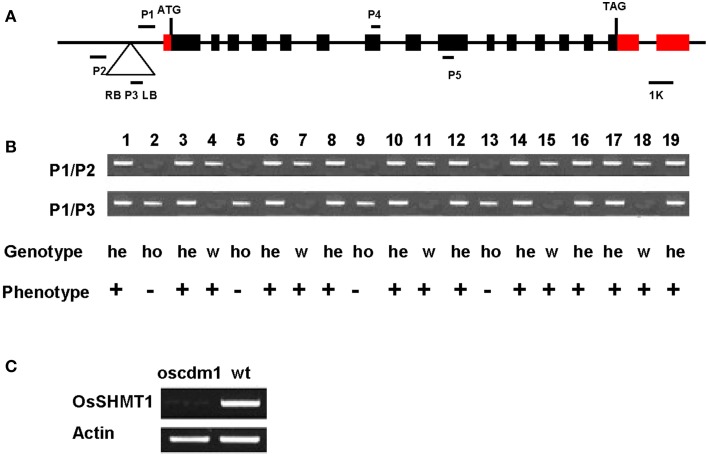
**Co-segregation of the T-DNA with the** ***chlorina*** **phenotype. (A)** Schematic representation of the T-DNA insertion upstream of *OsSHMT1* (exons = black/red boxes; introns = black lines) and the domain organization of OsSHMT1 (http://www.uniprot.org/uniprot/Q6EPF0). ATG, start codon; TAG, termination codon; RB, right border; LB, left border. P1, P2, and P3 are the primers used for genotyping. **(B**) Co-segregation analysis of the genotype and phenotype in a segregating population. All plants homozygous for the T-DNA insertion showed a chlorotic phenotype, indicating that the recessive mutation was caused by T-DNA insertion. Numbers represent the different plants tested. P1/P2, PCR using primers P1 and P2; P1/P3, PCR using primers P1 and P3; He, hemizygous; Ho, homozygous; W, wild type. “+” represents the wild-type phenotype; “−” represents the mutant phenotype. Plants 2, 5, 9, and 13 were homozygous because only the 0.9-kb band was amplified using primers P1 and P2. Plants 4, 7, 11, 15, and 18 were designated as wild type because only the 0.7-kb band was amplified using primers P1 and P3. In contrast, both the 0.7- and 0.9-kb bands were amplified from plants 1, 3, 6, 8, 10, 12, 14, 16, 17, and 19, indicating that they were heterozygotic. **(C)** Analyses of OsSHMT1 expression in wild-type and *oscdm1* mutant plants using actin as a control. The RT-PCR primers P4 and P5 for *OsSHMT1* span introns 7 and 8. RT-PCR using RNA from homozygous *oscdm1* plants did not detect full-length *OsSHMT1* transcripts.

T_2_ and T_3_ seedlings were genotyped to test whether the chlorotic phenotype co-segregated with the T-DNA insertion (Figures 4A,B). Two randomly selected lines, 4 and 18, exhibited a wild-type phenotype, and T_3_ seedlings of those lines were wild type in appearance. However, the progeny of plants 3 and 19 exhibited wild-type and mutant phenotypes at a ratio of 3:1. In contrast, plants 4 and 7 displayed the *chlorina* phenotype. These results demonstrate that the *chlorina* phenotype co-segregated with the T-DNA insertion. Furthermore, RT-PCR using RNA from the homozygous *oscdm1* plants did not detect full-length transcripts for *OsSHMT1*. Thus, the *oscdm1* plants were deemed to be loss-of-function mutants (Figure 4C).

### Functional complementation of *osshmt1*

**Figure 5:**
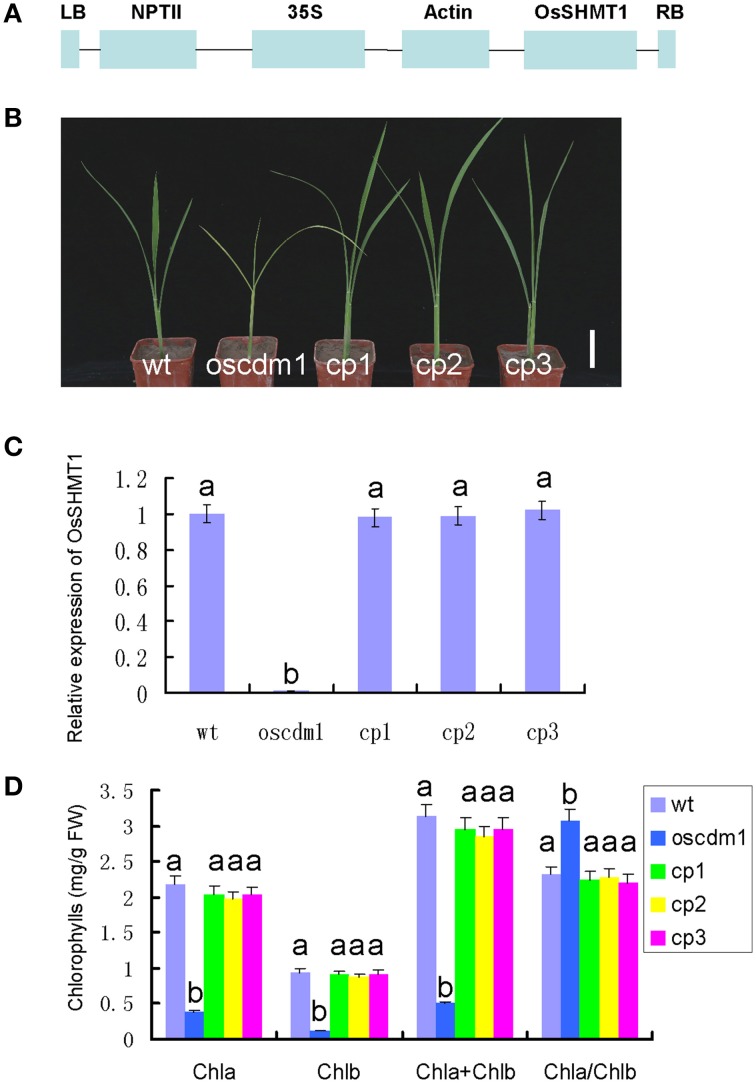
**Complemented expression of** ***OsSHMT1*** **rescued the mutant phenotypes of** ***oscdm1*****. (A)** Schematic structure of the actin-OsSHMT1 complementation vector. NPTII was used as a marker in the transformation test. **(B)** Complementation of the *oscdm1* mutant by the wild-type gene. *Japonica* rice *oscdm1* callus was used for transformation (see the “Materials and Methods”). Images of the wild-type plants, *oscdm1* mutant plants, and independent complemented plants (cp)1, cp2, and cp3 were taken after 1 month. Bar = 2 cm. **(C)** qRT-PCR analyses of *OsSHMT1* expression in wild type, *oscdm1*, and in cp1, cp2, and cp3 showed that the expression level in cp1, cp2, and cp3 was near the wild-type level. The experiments are biological replicates with the SD. **(D)** The leaf pigment contents in wild type, *oscdm1*, and in cp1, cp2, and cp3 were measured. The pigment contents of the complemented lines were rescued to the wild-type level. Data were compared by one-way analysis of variance and Duncan's multiple range test. Different letters (a–b) indicate significant differences (P, 0.05) between lines.

To further demonstrate that the *oscdm1* phenotype was due to insertion of the T-DNA into the candidate gene, functional complementation of the *oscdm1* mutant with wild-type *OsSHM*T1 was performed. Given the lethality of the homozygous mutation, homozygous mutant individuals were identified by genotyping the progeny of a heterozygous plant that was allowed to self-fertilize. These homozygous mutant individuals were used to initiate a callus in tissue culture, which was used for Agrobacterium-mediated transformation with the complementation construct. The sequence of *OsSHMT1* was used to search for the full-length cDNA sequence in the KOME database (http://cdna01.dna.affrc.go.jp/cDNA/). Fortunately, the full-length cDNA sequence of *OsSHMT1* was available. We cloned the full-length cDNA sequence by RT-PCR. The full-length cDNA sequence of *OsSHMT1*, which is 1542 bp in length, was found to be identical to that of the annotated gene Os03g0738400 in the TIGR database. This cDNA was cloned into a binary overexpression vector containing the actin promoter and subsequently transformed into *oscdm1* calli via *A. tumefaciens*-mediated transformation with G418 selection resistance (Figure 5A).

The resulting G418-resistant lines were confirmed using specific PCR primers (p1, p2, and p3) and cDNA-amplifying primers (OP1 and OP2). In addition, quantitative PCR revealed that the exogenous *OsSHMT1* gene was overexpressed in 50 transgenic lines (CP1, CP2, and CP3) (Figure 5C). Interestingly, these 50 lines displayed a wild-type phenotype even though their background was homozygous *oscdm1* (Figure 5B). The chlorophyll content in these lines was rescued to the wild-type level (Figure 5D).

### RNAi against *OsSHMT1* reproduced the *oscdm1* mutant phenotype

**Figure 6:**
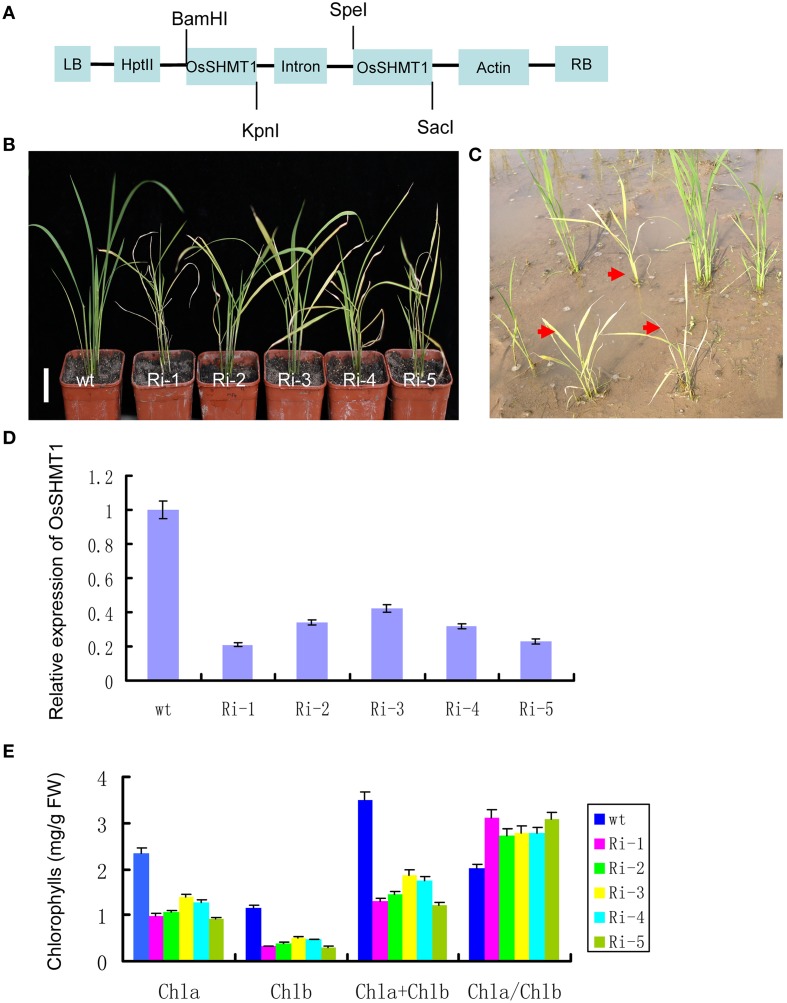
**RNAi against** ***OsSHMT1*** **in wild type mimicked the mutant phenotypes of** ***oscdm1*****. (A)** Schematic structure of the pUbi-RNAi309-SHMT1 vector, including the inverted repeat at the 3′ end of the SHMT DNA sequence. **(B)** Phenotypes of 1-month-old wild-type plants and five independent RNAi lines (Ri-1–5). Bar = 2 cm. **(C)** Photographs of independent RNAi lines in the paddy field after 40 days. **(D)** qRT-PCR analyses of OsSHMT1 expression in wild type (WT-1 and -2) and five independent RNAi lines (Ri-1–5). The results indicate that the expression of OsSHMT1 in the five independent RNAi lines had decreased to some extent. The experiments are biological replicates with the SD. **(E)** The pigment contents in the leaves of wild-type plants and independent complemented lines (Ri-1–5) were measured.

To further confirm that *OsSHMT1* was the gene associated with the observed mutant phenotype, an *OsSHMT1* RNAi vector was constructed and transformed into *japonica* variety Nipponbare (Figure 6A). About 400 independent transgenic lines were obtained and confirmed by PCR, and more than 100 of them displayed visibly chlorotic leaves during the early growth period (Figure 6B); however, after further growth and development the plants eventually died. Real-time PCR revealed that *OsSHMT1* transcription was decreased in the RNAi transgenic plants compared to that in the Nipponbare controls (Figure 6D). The chlorophyll contents were decreased to 11 and 37% of that in Nipponbare using five representative T_0_ lines (RNAi-1–5, respectively) (Figure 6E). Those RNAi plants showing a severe chlorotic phenotype eventually died in the field (Figure 6C). Conversely, other transgenic lines lacking the pUbi-RNAi309-SHMT1 vector (data not shown) failed to exhibit the chlorotic phenotype. These complementation and RNAi results strongly indicate that Os03g0738400 corresponds to *OsSHMT1*.

### Sequence analysis of *OsSHMT1*

BLAST searches of all available genomic sequences revealed that the rice genome contains five genes encoding an SHMT protein with strong homology to *OsSHMT1*. Close homologs of *OsSHMT1* were also identified in soybean and *Arabidopsis*. However, the functions of these genes are largely unknown. Notably, *OsSHMT1* and the *Arabidopsis* protein AtSHM1 (encoded by At4g37930) were found to possess a similar domain structure and to share the highest observed sequence homology across their entire lengths (87% amino acid identity). A neighbor-joining phylogenetic tree analysis based on the full-length protein sequences of SHMT family members from rice, soybean, and *Arabidopsis* showed that all of the SHMT protein sequences had a conserved pyridoxal phosphate binding site (Figures S2, S4).

**Figure 7:**
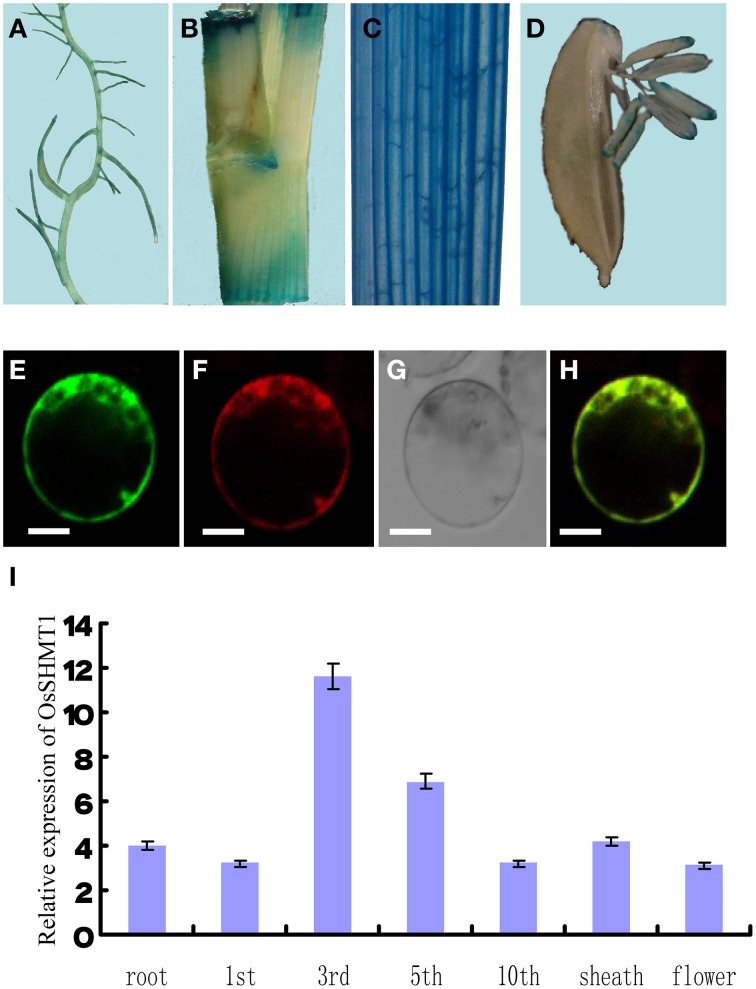
**The expression pattern and subcellular localization of OsSHMT1**. Different tissues were stained for GUS activity: **(A)** root; **(B)** internode; **(C)** blade; and **(D)** flower. The subcellular localization of OsSHMT1 was revealed in rice protoplasts as follows. **(E)** Subcellular localization of the OsSHMT1-GFP fusion protein in rice protoplasts. **(F)** MitoTracker® Red. **(G)** Bright field image of OsSHMT1-GFP. **(H)** Merged image of **(E,F)**. Bars = 5 μm. **(I)** Quantitative PCR was performed to analyze the expression of OsSHMT1 in root, first leaf, third leaf, fifth leaf, tenth leaf, sheath (mature leaf), and flower.

OsSHMT1 contains an apparent mitochondrial targeting sequence of 65 amino acid residues at its N-terminus. To test whether OsSHMT1 is localized to mitochondria, the complete *OsSHMT1* coding region was fused to the gene encoding GFP under the control of the CaMV 35S promoter. Vectors to express the 35S::OsSHMT1-GFP fusion protein and 35S::GFP (as a control) were introduced into rice protoplasts. MitoTracker® Red was used as a positive control for mitochondria. The 35S::OsSHMT1-GFP fusion protein co-localized with the MitoTracker® Red signal (see the merged image), suggesting that OsSHMT1 is a mitochondrial protein (Figures 7E–H).

### *OsSHMT1* expression pattern

An analysis by qRT-PCR revealed the expression of *OsSHMT1* mainly in young leaves, and not in root, leaf sheath, or flower, implying that *OsSHMT1* functions in seedlings or young leaves. To investigate the expression pattern of the gene in greater detail, total RNA was extracted from leaves from the first leaf stage to the mature stage. A qRT-PCR analysis of these RNA samples showed low-level expression of *OsSHMT1* at the first leaf stage; however, the expression level increased with leaf development and reached its highest level at the third leaf stage. Expression of the gene remained at a relatively high and stable level until the fifth leaf stage, suggesting that *OsSHMT1* is expressed at an early stage of growth and may play an important role in leaf development (Figure 7I).

Because the GUS gene in the enhancer trapping vector and the *OsSHMT1* gene shared the same orientation, we expected that the expression pattern in *oscdm1* would be consistent with the *OsSHMT1* expression pattern. Thus, we analyzed heterozygous plant tissues from an *oscdm1* segregating population for GUS staining. In vegetative shoots, strong GUS activity was noted in young leaf and leaf ligule, whereas relatively weak GUS activity was detected in root and flower. This result indicates that *OsSHMT1* is expressed mainly in young leaves, consistent with our qRT-PCR results (Figures 7A–D,I).

### Comparison of chlorophyll fluorescence parameters between wild-type and *oscdm1* plants

**Figure 8:**
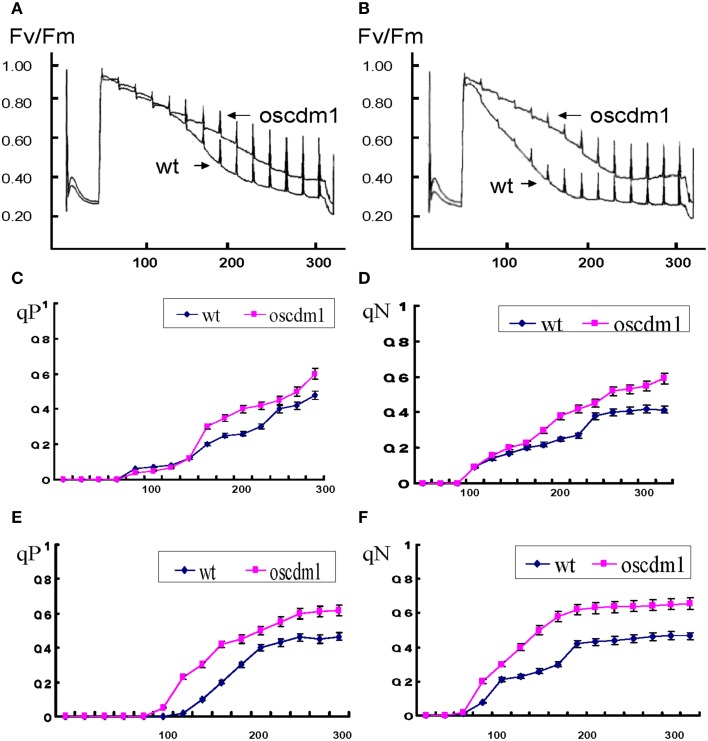
**Chlorophyll fluorescence analysis in** ***oscdm1*** **and wild-type plants. (A)** Chlorophyll fluorescence induction curve was produced using third leaf stage plants under actinic light **(A)**, low light intensity [57 μmol/m^2^s]; **(B)**, high light intensity [103 μmol/m^2^s]. The induction curve did not differ between wild type and the mutant up to 120 s of treatment under a low light intensity, but after 120 s the wild-type plants exhibited a greater fluorescence quenching capacity than the *oscdm1* plants. Moreover, under a high light intensity this change occurred at an earlier time. **(C–F)** Changes in qP and qN in wild-type and *oscdm1* plants. **(C,D)**, qP and qN changed under a low actinic light intensity (57 μmol/m^2^s). **(E,F)**, qP and qN changed under a high actinic light intensity (103 μmol/m^2^s). The data show that qP and qN were not overly different between the wild-type and *oscdm1* mutant plants up to 120 s of treatment at a low light intensity. However, under a high light intensity the change occurred earlier. This result suggests that the photochemical and non-photochemical quenching capacities of the mutant were affected and that the reaction speed became slower, especially under the higher light intensity. The data are the means ± SD (*n* = 5). These experiments were repeated more than three times with similar results.

Chloroplasts are the organelles that perform photosynthesis in plants; thus, they play an essential role in plant growth. To test whether the photosynthetic apparatus in the *oscdm1* plants was defective, we compared some key parameters of PSI and PSII between *oscdm1* and wild-type plants using an IMAGING-PAM fluorometer. The results of the comparison are shown in Table 2. Leaves from wild-type and *oscdm1* plants were dark-adapted for 10 min prior to fluorescence recording, and then a saturating light pulse was applied. F_v_/F_m_ is widely considered to be a sensitive indicator of the maximum quantum efficiency of PSII. The maximum quantum yield (F_v_/F_m_) of PSII in the *oscdm1* plants was ~0.6, which is considerably lower than the value of 0.83 found in wild-type plants under a low light intensity, and which is the normal value for the mesophyll cells of most plant species. During illumination with actinic light, the maximal fluorescence yields (F′_*m*_) were assessed in a time-dependent manner. The results indicate that the PSII quantum yield ratio [△ F/F′_*m*_ = (F′_*m*_−F)/F′_*m*_] was remarkably low in the *oscdm1* plants as compared with wild-type plants (Figures 8A,B). These results indicate that the *oscdm1* plants exhibited low photosynthetic activity because of damage to the reaction center of PSII.

**Table 2:** **Comparison of fluorescence parameters between wild-type (WT) and** ***oscdm1*** **rice leaves** (***n*** **= 15), the asterisks shows significantly different between the oscdm1 mutant and wild type at** ***P*** **≤ 0.05**.

**Parameter**	**57 μmol/m^2^s**		**103 μmol/m^2^s**	
	**WT**	***oscdm1***	**WT**	***oscdm1***
F_v_/F_m_	0.83 ± 0.06	0.60 ± 0.05^*^	0.82 ± 0.08	0.53 ± 0.04^*^
Electron transport rate	6.21 ± 0.31	0.52 ± 0.04^*^	6.15 ± 0.25	0.25 ± 0.05^*^
Yield	0.73 ± 0.11	0.35 ± 0.06^*^	0.68 ± 0.14	0.15 ± 0.03^*^

### The *oscdm1* mutant was more sensitive at a high light intensity

To detect the influence of different light intensities on the photosynthetic efficiency of the plants, *oscdm1* and wild-type plants were grown at a low (57 μmol/m^2^s) or high light intensity (103 μmol/m^2^s) and a chlorophyll fluorescence response curve was produced. Our results demonstrate no significant change in qP and qN between the wild-type and mutant plants up to 120 s of treatment at a low light intensity. However, at a high light intensity the change occurred earlier. This result suggests that the photochemical and non-photochemical quenching capacities of the mutant were affected, and that the reaction speed became slower, especially at a high light intensity. Similarly, a large change in qN was noted in the mutant plants at a high light intensity. The above results further demonstrate that the photosynthetic capacity of the mutant was abolished at a high light intensity (Figures 8C–F).

### *Oscdm1* is a photorespiratory mutant with a chlorotic phenotype caused by excessive H_2_O_2_

**Figure 9:**
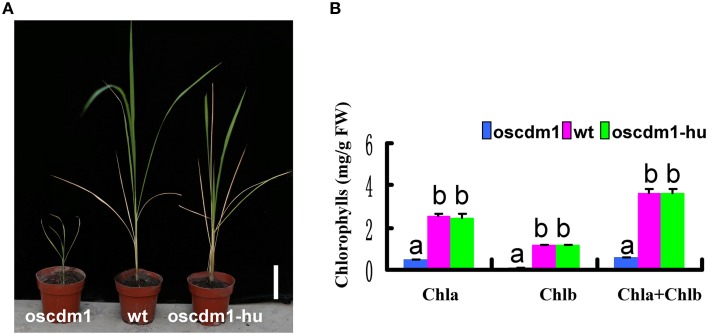
**The** ***oscdm1*** **mutant exhibited a wild-type phenotype under a high CO_2_ concentration. (A)** The *oscdm1* mutant (leftmost plant in **A**) was grown at an ambient CO_2_ level. The wild-type and *oscdm1*-hu plants (two rightmost plants in **A**) were grown under elevated CO_2_ levels in an artificial climate chamber. The photographs were taken after about 2 months. Bar = 5 cm. **(B)** The pigment contents of leaves from wild-type, *oscdm1*, and *oscdm1*-hu plants were measured. The pigment contents of the *oscdm1*-hu plants were restored to the wild-type level. The data represent the means ± SD. Bars represent the SDs (*n* = 10). Chla, chlorophyll *a*; Chlb, chlorophyll *b*. Data were compared by one-way analysis of variance and Duncan's multiple range test. Different letters (a–b) indicate significant differences (P, 0.05) between lines.

The *Arabidopsis* mutant *shm1-1* was one of the first photorespiratory mutants to be identified. The affected gene in *shm1-1* encodes an SHMT isozyme. The *shm1-1* mutant displays a lethal photorespiratory phenotype when grown at ambient CO_2_ levels, but it is virtually unaffected by elevated CO_2_ levels (Somerville and Ogren, 1981; Moreno et al., 2005; Jamai et al., 2009). Given the strong homology between *OsSHMT1* and *AtSHM1*, we examined whether the *oscdm1* mutant could survive exposure to a high (1%) CO_2_ concentration. Thus, *oscdm1* mutant (leftmost plant in Figure 9A) plants were grown under ambient CO_2_ levels while wild-type and *oscdm1*-hu (two rightmost plants in Figure 9A) plants were grown under elevated CO_2_ levels in an artificial climate chamber. After 1 month at 1% CO_2_, the *oscdm1* mutant plants were able to grow as well as wild type under ambient conditions (Figure 9A). Since the plants were kept in an artificial climate chamber for an extended period, some senescing leaves were observed on the wild-type and *oscdm1* plants. The pigment content of the *oscdm1*-hu plants was restored to the wild-type level (Figure 9B). This result is similar to that obtained for *Arabidopsis shm1-1*.

**Figure 10:**
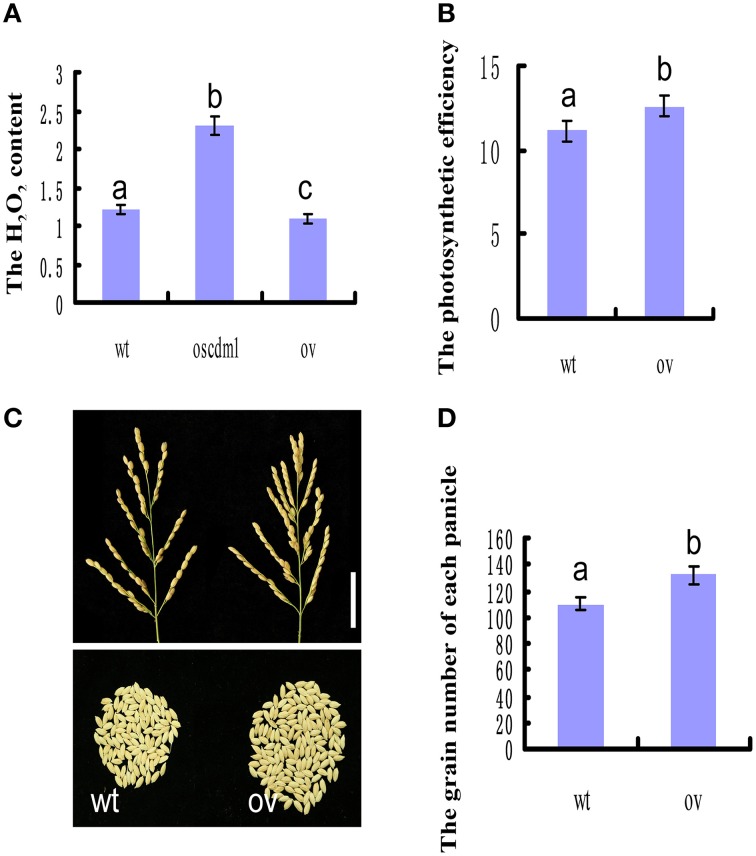
**OsSHMT1-OV plants exhibited improved photosynthetic efficiency and productivity. (A)** The H_2_O_2_ contents were measured among wild-type, *oscdm1* mutant, and OsSHMT1-OV plants at the fourth leaf stage. **(B)** The photosynthetic efficiency was measured between wild-type and OsSHMT1-OV plants at the mature stage. **(C)** Comparison of wild-type and OsSHMT1-OV panicles. **(D)** Comparison of the grain number per panicle between wild-type and OsSHMT1-OV panicles. The data are the means ± SD (*n* = 5). These experiments were repeated more than three times with similar results. Data were compared by one-way analysis of variance and Duncan's multiple range test. Different letters (a–c) indicate significant differences (P, 0.05) between lines.

Given that the *Arabidopsis* mutant *atshmt1-1* accumulates reactive oxygen species (ROS) under natural conditions (Moreno et al., 2005), we investigated the H_2_O_2_ contents among wild-type, *oscdm1* mutant, and OsSHMT1-OV plants. As shown in Figure 10A, for all of the lines the H_2_O_2_ level in the *oscdm1* mutant plants was significantly higher than that in wild type at the fourth leaf stage. By contrast, the level of accumulation of H_2_O_2_ in the OsSHMT1-OV transgenic lines was slightly lower than that in wild type (Figure 10A). The above analyses indicate that *OsSHMT1* encodes an SHMT that is required for photorespiration and which plays a role in attenuating ROS production in chloroplasts to mitigate photoinhibition.

### The OsSHMT1-OV transgenic lines exhibited increased photosynthetic efficiency and improved productivity

To evaluate whether *OsSHMT1* could improve photosynthetic efficiency, several photosynthetic parameters were measured using a portable LCPRO^+^ photosynthesis measurement instrument (ADC BioScientific). The OsSHMT1-OV transgenic lines had a higher photosynthetic rate, stomatal conductance, intercellular CO_2_ concentration, and transpiration rate than wild type at the heading stage (Figure 10B and Table 3). Interestingly, the productivity (grain number per plant) of the OsSHMT1-OV lines was 5% greater than that of wild type (Figures 10C,D). Thus, OsSHMT1 overexpression can improve the photosynthetic efficiency of plants and could be applied to breeding programs in the future.

**Table 3:** **Comparison of photosynthesis parameters between WT and OsSHMT1-OV rice leaves** (***n*** **= 15), the asterisks shows significantly different between the OsSHMT1-OV and wild type at** ***P*** **≤ 0.05**.

**Parameter**	**WT**	**OsSHMT1-OV lines**
Stomatal conductance (mol/m^2^s)	0.159 ± 0.03	0.194 ± 0.05^*^
Intercellular CO_2_ concentration (μmol/mol)	237.1 ± 5.2	333.6 ± 7.3^*^
Transpiration rate (mmol/m^2^s)	1.77 ± 0.21	2.62 ± 0.25^*^

## Discussion

### Enhancer trapping is a valuable tool for gene identification

GAL4/VP16-UAS, the improved binary T-DNA vector used to construct our study population (Wu et al., 2003; Peng et al., 2005), contains a promoter-less GUS reporter gene next to the RB. This enhancer trapping vector is designed to detect fusion between the GUS gene and an endogenous gene tagged by the T-DNA. Insertion of the construct not only destroys normal gene function, it also activates expression of the reporter gene. In rice, at least 40% of the insertion events led to GUS activation in various tissues (e.g., roots, leaves, flowers, and seeds) (Peng et al., 2005). Since T-DNA insertion lines do not all carry a single copy of the insert, positive GUS staining results do not fully confirm co-segregation between the mutant phenotype and gene. Still, this method has a number of advantages. First, it can be used to identify a candidate gene from several mutant lines in a short amount of time. Jung et al. (2003) used gene trapping technology to clone *OsCHLH*; since then, a number of genes, including *Wda1* (Jung et al., 2006), have been successfully cloned using this method. Second, enhancer trap vectors can be used to determine candidate gene expression patterns. GUS expression data can provide helpful information for functional gene analyses, including spatial-temporal expression specificity and clues as to a gene's function (Johnson et al., 2005; Peng et al., 2005; Liang et al., 2006). The cell division-related gene *CYCD3;2* was successfully cloned by reporter gene trapping. The GUS staining result in that case was similar but slightly different from the temporal patterns in T-DNA lines owing to the opposing orientation of the T-DNA insertion and *CYCD3;2*. This result may have been influenced by neighboring genes (Swaminathan et al., 2000). In the present study, the GUS gene was in the same orientation as *OsSHMT1*. Thus, the technique provided accurate *OsSHMT1* organ expression patterns and yielded helpful data for our bioinformatic analysis and prediction of gene function.

### The *OsSHMT1* gene has a conserved regulatory function in monocots and dicots

In this report, a leaf color mutant with a *chlorina* phenotype was identified from a rice T-DNA-tagged pool and the gene was trapped by GUS staining. The mutated gene was identified as *OsSHMT1*, which encodes the largest subunit of SHMT. Rice OsSHMT1 is highly homologous to SHMT from *Arabidopsis*. The T-DNA was integrated into the *OsSHMT1* promoter, 99 bp away from the start codon (ATG). This generated a fusion transcript between the *OsSHMT1* promoter and GUS. Moreover, because of the insertion, the wild-type *OsSHMT1* transcript was not detectable in plants homozygous for the T-DNA. T-DNA insertion led to a loss-of-function mutation in *OsSHMT1*. The rice genome includes five genes that are homologous to *OsSHMT1*. RNAi against four other SHMT family genes caused no change in photorespiration compared with wild type (data not shown). This suggests that *OsSHMT1* is only one of multiple photorespiration-related genes in the rice SHMT family. SHMT in *Arabidopsis* is encoded by seven *SHM* genes, two of which encode mitochondrial isoforms (Jamai et al., 2009). However, only *SHM1* is necessary and sufficient to specify photorespiratory SHMT activity (Voll et al., 2006). The *oscdm1* plants produced chlorotic leaves, accumulated excessive H_2_O_2_, and subsequently died, similar to the mutant *Atshm1*. Moreover, the *oscdm1* plants were able to survive at a high concentration of CO_2_ (1% CO_2_). Therefore, SHMT family genes appear to have a conserved regulatory function in photorespiration in both monocots and dicots.

### The *oscdm1* mutant exhibits damage caused by photooxidation

Leaf color mutants may result from a variety of problems, including defects in chlorophyll synthesis, abnormal degradation pathways, and light damage. The *oscdm1* mutant is different from other reported leaf color mutants of rice. Almost all leaf color mutants show the mutant phenotype at the start of the second to the third leaf stage; at that stage, the *oscdm1* plants began to show slight chlorosis, which increased gradually. Structurally, the chloroplasts in the *oscdm1* plants developed well initially, but as time progressed the chloroplasts were destroyed and the plants eventually died. This chain of events is similar to that observed for photorespiration mutants of *Arabidopsis*. Photorespiration dissipates excess light energy as a protective process in plants. Based on our results, the phenotype of the *oscdm1* plants was due to photooxidation and changes in chloroplast structure. When the leaves of the mutant were subjected to stress, the chloroplasts exhibited ultrastructural changes, including irregularly stacked grana, and symptoms of vacuolization. The ultrastructural changes observed in the chloroplasts of the *oscdm1* plants are similar to the ultrastructural changes observed in the leaves under conditions of stress. When the plants were under stress, the level of ROS increased and caused cellular injury due to membrane oxidation. A number of physiological processes were affected, including photosynthesis, programmed cell death, hormone action, growth and development, and mitochondrial membrane structure (Figure S5). In higher plants, multiple SHMT genes predicted to localize to different compartments have been described (Zhang et al., 2010). Apart from its photorespiratory role in mitochondria, the physiological roles of SHMT are not well characterized. Given the finding that the OsSHMT1-OV lines had a lower H_2_O_2_ concentration compared with wild type, *OsSHMT1* may participate in biotic and abiotic stress responses. Genetic engineering using *OsSHMT1* represents a potential way to improve the tolerance of crop plants to stress.

### Differences between the current study and a recently published report

As we were submitting the present study for review, a report entitled “Characterization and molecular cloning of a serine hydroxymethyltransferase 1 (OsSHM1) in rice” in the *Journal of Integrative Plant Biology* (Wang et al., 2015). Both studies indicate that OsSHM1 is involved in photorespiration in rice, and that it has a conserved function in dicots and monocots. However, although the two mutants are allelic, there are many differences between the two studies. First, the *OsSHMT1* gene was cloned by two different methods: T-DNA tagging in our study and map-based cloning in the study of Wang et al. The corresponding mutant exhibited a loss-of-function point mutation (a single nucleotide change [T1300C] in LOC_Os03g52840); however, for further analysis of the function of *OsSHMT1*, the *oscdm1* mutant obtained in the present study offers several advantages over the mutant described by Wang et al. The mutant oscdm1 not only provided a good material for photorespiration, but also *OsSHMT1* specical enhancer and promoter was further studied by oscdm1 mutant. Second, several additional color mutants and genes were isolated and presented in our study, including SOD (A167) and GTP (A401-1). Thus, the present report provides significant information and materials for further analysis of SOD (A167) and GTP (A401-1) function. Third, a greater number of photosynthetic parameters were measured in this study vs. that of Wang et al. In particular, the use of different light intensities and BN gel analysis in this study suggested that thylakoid membrane proteins (e.g., LHCs) were disrupted, extending the finding that photosynthesis was impaired. Fourth, and perhaps most interesting and/or valuable, our OsSHMT1-OV transgenic lines exhibited increased photosynthetic efficiency and improved productivity. These lines will be of great value for breeding high-efficiency cultivars of rice and other species.

### The OsSHMT1-OV lines exhibited increased photosynthetic efficiency and improved productivity

A particularly intriguing finding of our study is that *OsSHMT1* transcript accumulation increased the photosynthetic efficiency and productivity of the plants. This phenomenon can be explained as follows. The negative impact of photorespiration on plant growth and yield has been demonstrated by doubling the CO_2_ concentration in a glasshouse environment; it can dramatically increase the performance of several crops. However, such a strategy cannot be implemented in the field, where photorespiratory losses are exacerbated by high temperatures and suboptimal water supplies. Under these conditions, the stomata close and the intercellular O_2_ concentration increases through the release of O_2_ from the light reactions of photosynthesis. Despite these disadvantages, photorespiration is important because it recovers 75% of the carbon from phosphoglycolate and efficiently removes potent inhibitors of photosynthesis. Moreover, photorespiration dissipates excess photochemical energy under high light intensities, thus protecting chloroplasts from overreduction. Kebeish et al. (2007) introduced the *Escherichia coli* glycolate catabolic pathway into *A. thaliana* chloroplasts to reduce the loss of fixed carbon and nitrogen that occurs in C3 plants when phosphoglycolate, an inevitable by-product of photosynthesis, is recycled by photorespiration. In our study, the transgenic OsSHMT-OV lines grew faster, produced more shoots and a greater panicle biomass, and contained more soluble sugars, reflecting reduced photorespiration and enhanced photosynthesis that correlated with an increased chloroplastic CO_2_ concentration in the vicinity of ribulose-1,5-bisphosphate carboxylase/oxygenase. The greater panicle biomass of the plants indicates that the overexpression of *OsSHMT1* enabled the plants to overcome at least some of the photorespiration-induced limitations on growth. This result indicates that the increase in biomass production of the transgenic plants coincided with improved photosynthesis.

In conclusion, we showed that the oscdm1 chloroplast thylakoid membrane damage may be from the excessive reactive oxygen (H_2_O_2_). The reactive oxygen (H_2_O_2_) was mainly produced in mitochondria. Mitochondria and chloroplasts are thought to have originated from prokaryotes during endosymbiotic evolution in eukaryotic cells. Intimate communication among organelles is necessary to coordinate their activities during growth, development, and other physiological processes. It is reasonable to assume that an intracellular mobile signal is transported from mitochondria to chloroplasts to trigger chloroplast change. This signal should be clarified in future investigations by taking advantage of *oscdm1* plants.

### Conflict of interest statement

The authors declare that the research was conducted in the absence of any commercial or financial relationships that could be construed as a potential conflict of interest.

## Supplementary material

Figure S1 **Schematic map of the enhancer trap vector**. The upper and lower portions of the map show the T-DNA and vector backbone, respectively. LB, left border of the T-DNA; RB, right border of the T-DNA. Additional key functional elements are shown in this map. −48CaMV, which was activated by chromosomal enhancer elements, is a minimal CaMV 35S promoter. *GAL*4/*Vp*16 is a modified gene composed of the yeast transcriptional activator GAL4 DNA-binding domain and herpes simplex virus *VP*16 activation domain, fused to the minimal promoter –48CaMV. The reporter gene is also a fused gene containing BoGUS and EGFP. 6×UAS, an upstream activator sequence with six repeats, was located in front of the reporter gene.

Figure S2 **Sequence alignment of SHMT homologs from soybean, rice, and** ***Arabidopsis***. Identical residues are boxed in black; similar residues are highlighted in gray. The pyridoxal phosphate binding site is marked using a red line. The OsSHMT1 SHMT domain spans residues 51–449.

Figure S3 **BN gel analysis of thylakoid membrane protein complexes**. Lane l, wild type; lane 2, *oscdm1*. Band I, PSI monomer and PSII dimer; II, ATP synthase; III, monomer; IV, CP43-less PSII; V, LHC II.

Figure S4 **A phylogenetic tree showing the predicted relationships among soybean, rice, and** ***Arabidopsis***. The full-length amino acid sequences of each protein were aligned using CLUSTALW and revised manually. The tree was constructed using the neighbor-joining method with full-length protein sequences from soybean, rice, and *Arabidopsis*. The results indicate that the SHMT protein sequences were divided into three subfamilies: SUBI [*Oryza sativa*: OsSHMT1 and OsSHMT3; soybean: GmSHMT1 and GmSHMT4; and *Arabidopsis*: AtSHMT3, AtSHMT4, and AtSHMT5]; SUBII [*Oryza sativa*: OsSHMT2 and OsSHMT4; soybean: GmSHMT3 and GmSHMT5; and *Arabidopsis*: AtSHMT1 and AtSHMT2]; and SUBIII [*Oryza sativa*: OsSHMT5; soybean: GmSHMT2; and *Arabidopsis*: AtSHMT6 and AtSHMT7]. The bar represents the genetic distance in the phylogenetic tree.

Figure S5 **Transmission electron microscopic images of mitochondria from 1-month-old wild-type and** ***oscdm1*** **mutant plants. (A)** A wild-type plant. **(B)** An *oscdm1* plant. The wild-type mitochondrial membranes were well-ordered, while the mutant mitochondrial membranes were abnormal and became irregular. Bar = 100 nm.

Table S1 **Primer sequence for test**.
