# Supplementary figures and images for: The molecular cloning and clarification of a photorespiratory mutant, oscdm1, using enhancer trapping
Source: Front Genet. 2015 Jul 3;6:226. doi: 10.3389/fgene.2015.00226 (PMC4490251; doi:10.3389/fgene.2015.00226)

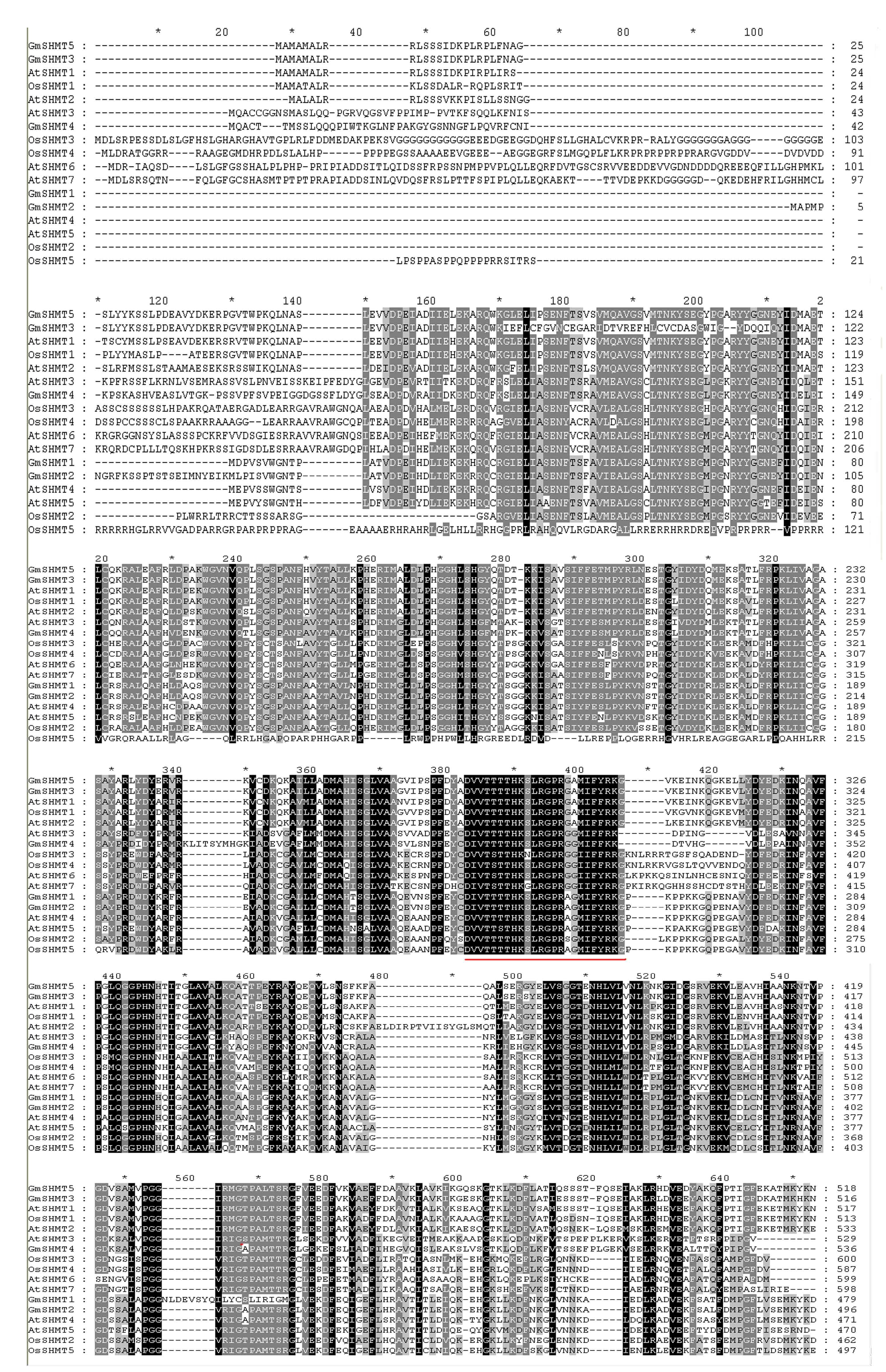

Supplement: Figure S2 — Sequence alignment of SHMT homologs from soybean, rice, and Arabidopsis. Identical residues are boxed in black; similar residues are highlighted in gray. The pyridoxal phosphate binding site is marked using a red line. The OsSHMT1 SHMT domain spans residues 51–449. [file Image2.TIF]

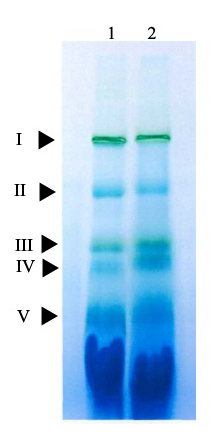

Supplement: Figure S3 — BN gel analysis of thylakoid membrane protein complexes. Lane l, wild type; lane 2, oscdm1. Band I, PSI monomer and PSII dimer; II, ATP synthase; III, monomer; IV, CP43-less PSII; V, LHC II. [file Image3.TIF]

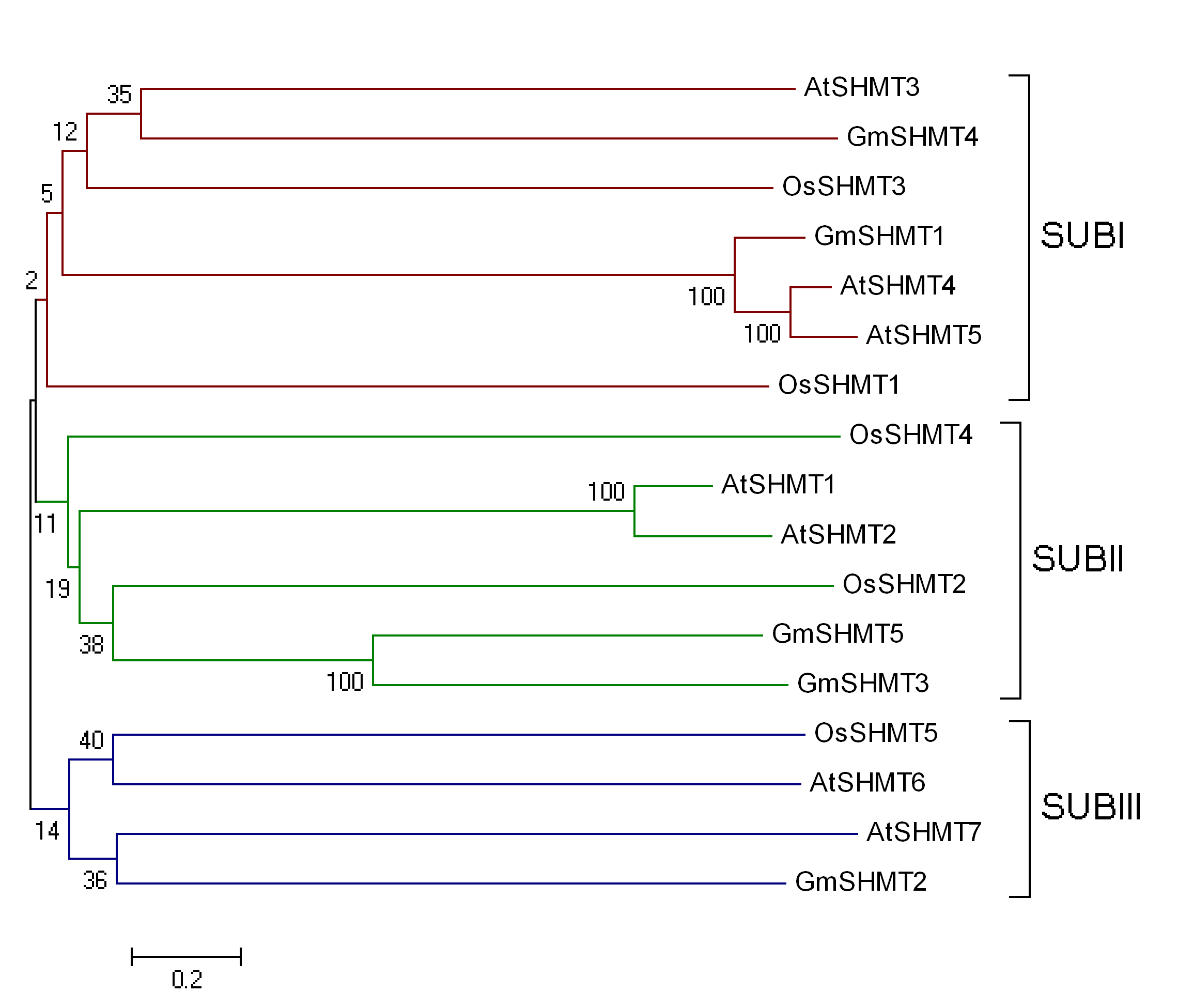

Supplement: Figure S4 — A phylogenetic tree showing the predicted relationships among soybean, rice, and Arabidopsis. The full-length amino acid sequences of each protein were aligned using CLUSTALW and revised manually. The tree was constructed using the neighbor-joining method with full-length protein sequences from soybean, rice, and Arabidopsis. The results indicate that the SHMT protein sequences were divided into three subfamilies: SUBI [Oryza sativa: OsSHMT1 and OsSHMT3; soybean: GmSHMT1 and GmSHMT4; and Arabidopsis: AtSHMT3, AtSHMT4, and AtSHMT5]; SUBII [Oryza sativa: OsSHMT2 and OsSHMT4; soybean: GmSHMT3 and GmSHMT5; and Arabidopsis: AtSHMT1 and AtSHMT2]; and SUBIII [Oryza sativa: OsSHMT5; soybean: GmSHMT2; and Arabidopsis: AtSHMT6 and AtSHMT7]. The bar represents the genetic distance in the phylogenetic tree. [file Image4.TIF]
