# Supplementary material for: The molecular cloning and clarification of a photorespiratory mutant, oscdm1, using enhancer trapping
Source: Front Genet. 2015 Jul 3;6:226. doi: 10.3389/fgene.2015.00226 (PMC4490251; doi:10.3389/fgene.2015.00226)
Supplement: Table S1 — Primer sequence for test. [file Table1.DOC]

Table S1: Primer sequence for test.

| Primer Name | Primer Sequence |
| --- | --- |
| P1 | GGT TTG GGA GGT AGT GGA TTC G |
| P2 | TCC TGC CTC TGG GAC CAA CAT C |
| LB1 | CGA TGG CTG TGT AGA AGT ACT CGC |
| P3( LB2) | CGG TCA ATA CAC TAC ATG GCG TG |
| P4 | GCG CTT AAG CAG GCA ACT AC |
| P5 | CTG CAA TGT GCA CGT TTT CT |
| P6 | GGG TAC CAC TAG TTG GAG AAA AGT GCC GTT CTT |
| P7 | CGG GAT CCG AGC TCT GCT TAA GCG CAA CAG CTA A |
| OP1 | ACG CGT CGA CCA CCT CAG GAA TTC AGC TTG ATG |
| OP2 | TAC CCG GGT CGC CGC TCG CCC ACC AT |
| Q1f | ACG CGT CGA CCA CCT CAG GAA TTC AGC TTG ATG |
| Q1R | TAC CCG GGT CGC CGC TCG CCC ACC AT |
| Actinf | GAC CTT GCT GGG CGT GAT CTC |
| Actinr | GAT GGG CCA GAC TCG TCG TAC |
| ADAR1 | CTA ATA CGG TCA CTA TAG CGC TCG AGC GGC CGC CGG GGA GGT |
| ADAR2 | p-ACC TCC CCNH2 |
| APR1 | GGA TCC TAA TAC GAG TCA CTA TAG CGC |
| APR2 | CTA TAG CGC TCG AGC GGC |
| FP1 | TCTAGAATGGCCATGGCGACGG |
| RP1 | CCCGGGGTTCTTGTACTTCATGGTTTCTTTCT |
